# Smoking, mental illness and socioeconomic disadvantage: analysis of the Australian National Survey of Mental Health and Wellbeing

**DOI:** 10.1186/1471-2458-13-462

**Published:** 2013-05-11

**Authors:** David Lawrence, Jennifer Hafekost, Philip Hull, Francis Mitrou, Stephen R Zubrick

**Affiliations:** 1 Telethon Institute for Child Health Research, Centre for Child Health Research, The University of Western Australia, P.O. Box 855, West Perth, WA, 6872, Australia; 2 Cancer Council New South Wales, P.O. Box 572, Kings Cross, NSW, 1340, Australia

## Abstract

**Background:**

High rates of smoking and lower rates of smoking cessation are known to be associated with common mental disorders such as anxiety and depression, and with individual and community measures of socioeconomic status. It is not known to what extent mental illness and socioeconomic status might be jointly associated with smoking behaviour. We set out to examine the relationship between mental illness, measures of socioeconomic disadvantage and both current smoking and smoking cessation rates.

**Methods:**

We used data from the 2007 Australian National Survey of Mental Health and Wellbeing to examine the relationship between mental illness, socioeconomic status and both current smoking and smoking cessation. We used cross-classified tables and logistic regression to examine the relationship between psychosocial and sociodemographic predictors and current smoking. We also used proportional hazards regression to examine the relationship between the factors and smoking cessation.

**Results:**

Both mental illness and socioeconomic status were independently associated with current smoking and with lower likelihood of smoking cessation, with gradients in smoking by mental health status being observed within levels of socioeconomic indicators and vice versa. Having a mental illness in the past 12 months was the most prevalent factor strongly associated with smoking, affecting 20.0% of the population, associated with increased current smoking (OR 2.43; 95% CI: 1.97-3.01) and reduced likelihood of smoking cessation (HR: 0.77; 95% CI: 0.65-0.91).

**Conclusions:**

The association between mental illness and smoking is not explained by the association between mental illness and socioeconomic status. There are strong socioeconomic and psychosocial gradients in both current smoking and smoking cessation. Incorporating knowledge of the other adverse factors in smokers’ lives may increase the penetration of tobacco control interventions in population groups that have historically benefitted less from these activities.

## Background

There have been enormous declines in smoking rates in Australia and other developed countries since the 1950s [1-3]. There is substantial evidence to show that current smoking and lower rates of smoking cessation are associated with common mental health problems such as anxiety, depression and dependence on alcohol or other drugs, and are also associated with a range of indicators of socioeconomic disadvantage such as income, educational attainment, and measures of community disadvantage. For instance, it has been estimated that a third of current adult smokers in Australia and the United States have common mental disorders such as anxiety, depression and alcohol and drug use disorders [4,5]. Despite similar levels of wanting to quit, attempting to quit and similar levels of access to smoking cessation therapies, compared with other smokers, people with mental disorders have lower smoking cessation rates, smoke on average for longer durations and suffer increased morbidity and mortality as a result [6,7].

Both individual and area-level markers of socioeconomic status have been associated with smoking status [8-10]. Lower levels of socioeconomic status are associated with a range of lifestyle risk factors for poor health outcomes, lesser interest in lifestyle changes for health reasons, and reduced access to health care and health services [11,12]. Socioeconomic disadvantage is also associated with poorer mental health, and poor health outcomes in general [13,14].

Knowledge of the sociodemographic and psychosocial characteristics of smokers is helpful in planning future tobacco control activities. Tobacco control policies and programs have been very effective in reducing smoking rates in developed countries, but these programs have been most successful among more highly educated people, and people without other complicating factors such as mental illness [15-17]. Some have suggested that continued reductions in smoking rates will require adapting tobacco control policies to the specific challenges facing today’s smokers [17,18]. Recognition of the changing demographic among current smokers and the need for innovation in tobacco control to address this population is starting to occur with, for instance, the Australian National Preventative Health Taskforce proposing a variety of measures designed to target a range of population sub-groups known to be at high risk of smoking, including prisoners, homeless people, and people living in highly disadvantaged neighbourhoods [19]. Where programs and public health promotions have been designed to address the issue of mental illness, these have almost always focussed on severe mental illness such as schizophrenia, or people living in institutionalised settings or in receipt of specialised mental health treatment [20,21]. Reference to people with common mental disorders living in the community is noticeably absent from major tobacco control policy documents in Australia and the United States [19,22].

While a range of sociodemographic and psychosocial factors has been identified that are associated with current smoking and lower quit rates, it is unknown to what extent these factors interact. It is possible that the relationships observed between smoking and anxiety and depressive disorders merely reflect the higher prevalence of these disorders among population groups with lower levels of socioeconomic status. Alternatively the association with socioeconomic disadvantage may reflect the higher proportion of mental disorders in these groups. It is also plausible that both sets of factors are associated with smoking outcomes, and they may interact in some way.

Using a large population-based sample of Australian adults which assessed smoking status and history along with a comprehensive assessment of mental health status and measurement of a large range of individual and familial markers of socioeconomic status along with a small area measure of socioeconomic status, we examined the relationship between individual factors and both current smoking and smoking cessation rates, the degree to which these factors co-occur and their joint association with smoking status. We set out to test the null hypothesis that after adjusting for a comprehensive set of markers of socioeconomic disadvantage there would be no independent association between mental health status and smoking.

## Methods

### Data source

We used data from a large-scale population-representative sample of Australian adults aged between 16–85 years. The Australian National Survey of Mental Health and Wellbeing (NSMHWB) was conducted by the Australian Bureau of Statistics (ABS) between August and December 2007 [23,24]. It comprised a nationally representative sample of 8,841 adults aged 16–85 years living in private dwellings, based on a stratified multistage area-based sample design. The survey was conducted by means of personal interview in the home. While the principal aim of the survey was to measure the prevalence of three major groups of mental disorders — anxiety disorders, affective disorders and substance use disorders — the survey also collected a comprehensive set of sociodemographic indicators, and assessed smoking status and smoking history. Full details of the survey methodology have been published elsewhere [25,26].

As the study consisted of analysis of publicly available confidentialised files, no institutional ethics approval was required.

### Measures

#### Tobacco use

In the NSMHWB, respondents were asked, “have you smoked at least 100 cigarettes in your life?” and “do you currently smoke every day, at least weekly, less than weekly, or not at all?” Current smokers were asked, “at what age did you start smoking every day?” Non-current smokers were asked “have you ever smoked every day?” If so they were asked “at what age did you start smoking every day?” All former smokers were asked “at what age did you stop smoking every day?” Having smoked at least 100 cigarettes is a commonly used measure of being a lifetime ever smoker to rule out teenage experimentation. These questions were used to classify all survey participants as current smokers, former smokers or lifetime non-smokers. For current and former smokers, age started smoking and age ceased smoking for former smokers were used to analyse time to smoking cessation.

#### Mental illness

Mental disorders were assessed in the NSMHWB using Version 3 of the World Health Organization’s Composite International Diagnostic Interview (CIDI) [27]. The CIDI is a fully structured interview questionnaire which was administered by lay interviewers using computer assisted interviewing software. The CIDI is designed to cover the diagnostic criteria for mental disorders in both the International Classification of Diseases, 10th edition (ICD-10) [28], and the Diagnostic and Statistical Manual of Mental Disorders, 4th Edition (DSM-IV) [29]. The CIDI includes an initial screener for major symptoms of mental disorders followed by detailed questions on each disorder. The average interview time in the NSMHWB was 90 minutes, with the majority of the time taken up by the administration of the CIDI.

For the purposes of this report, ICD-10 diagnoses were employed, although similar results are found with the DSM-IV diagnoses. Presence of the following disorders was assessed in the survey: anxiety disorder (panic disorder, social phobia, agoraphobia, generalised anxiety disorder, post-traumatic stress disorder or obsessive-compulsive disorder), depressive disorder (depressive episode, dysthymia or bipolar affective disorder), or substance use disorder (alcohol harmful use, alcohol dependence, drug dependence). Both lifetime and past 12-month status were assessed.

### Indicators of socioeconomic status

#### Household income

Household income is derived from the income of all usual residents 15 years and older. For each resident the household spokesperson was asked, “before income tax is taken out how much does he/she receive from all sources in total?” Household incomes were grouped into quintiles.

#### Education

To determine highest level of educational attainment each respondent was asked, “what was the highest year of primary or secondary school you completed?” followed by, “what is the level of the highest qualification that you have completed?” These questions were combined to create a measure of educational attainment based on highest year of schooling completed for people with no post-school qualification, or otherwise based on level of post-school qualification.

#### Ever been homeless

All respondents were asked “have you ever been homeless?”

#### Ever been in gaol, prison or correctional facility

Respondents were asked, “were you ever in gaol, prison or a correctional facility?”

#### Registered marital status

Respondents were asked, “what is your marital status?” If they reported that they were in a de facto relationship, single or not married they were asked, “have you ever been in a registered marriage?” and if so, “are you widowed, divorced or separated?”

#### Tenure type

Tenure type was established through a series of questions to respondents. Respondents were asked, “is this dwelling being paid off by you [or your spouse/partner/parent]?” If no, they were asked, “is this dwelling owned outright by you [or your spouse/partner/parent]?” If no, they were asked, “is this dwelling rented by you [or your spouse/partner/parent]?” If no, they were asked, “is this dwelling being purchased under a rent/buy or shared equity scheme by you [or your spouse/partner/parent]?” If none of these questions were endorsed the respondent was asked, “do you [or your spouse/partner/parents] occupy this dwelling under a life tenure scheme?” If no, they were asked, “do you [or your spouse/partner/parents] pay board to live here?” and if no they were asked, “do you [or your spouse/partner/parents] live here rent free?”

#### Financial difficulties

To determine if the household has had financial difficulties respondents were asked, “over the past 12 months, have any of the following happened to (you/your household) because of a shortage of money?” Respondents were shown a list of seven items which included, “could not pay electricity, gas or telephone bills on time”, “could not pay for car registration or insurance on time”, “pawned or sold something”, “went without meals”, “unable to heat my home”, “sought assistance from welfare/community organisations” and “sought financial help from friends or family”. Households were classified as having experienced financial difficulties in the last 12 months if any of these items was endorsed.

#### Labour force status

To establish if respondents were employed, unemployed or not in the labour force they were asked, “last week did you do any work at all in a job, business or farm?” and if not, “did you have a job, business or farm that you were away from because of holidays, sickness or any other reason?”

#### Occupation of main job

Those respondents who were currently employed were asked, “what was your occupation in that job?”, “what were your main tasks or duties?”, “what kind of business or service is carried out by your employer in the place where you work?” and “what is the name of your employer?” Based on this information occupations were classified according to the Australian and New Zealand Standard Classification of Occupations [30].

#### Family composition of household

The family composition of the household was derived from all the people usually living in the household and the relationships between them. For each individual the household spokesperson was asked, “What is (your/PERSON’S) relationship to (HOUSEHOLD REFERENCE PERSON/you)?”

#### Main source of income

Respondents were asked to respond to the question, “what is your main source of income?” by selecting one of the following answers “profit or loss from own unincorporated business or share in a partnership”, “profit or loss from rental properties”, “dividends or interest”, “wages/salary (including from own unincorporated business)”, “government pension or allowance (include family tax benefit, if received as payment from centrelink)”, “child support or maintenance”, “superannuation or annuity”, “workers’ compensation” or “any other regular source”.

#### Relative socio-economic disadvantage

In addition to these individual and household measures, all respondent addresses were geocoded to the level of census collection districts (CCDs). These are small geographic areas averaging around 200–250 households in size. Based on data collected in the 2006 Australian Census of Population and Housing, all CCDs have been assigned an Index of Relative Socioeconomic Disadvantage. This is based on proportion of people living in that area with low income, low educational attainment, unemployment, and dwellings without motor vehicles [31]. All CCDs in Australia were ranked by this index and grouped into quintiles.

### Weighted estimates and standard errors

Survey weights were applied to calculate estimates of totals and proportions. These weights have been calculated to adjust for potential non-response. Standard errors and confidence intervals for the NSMHWB were calculated using the jack-knife method of replicate weighting [32]. For each indicator of socioeconomic status we calculated the proportion of the population that fell into each level of that indicator. Within each level we calculated the proportion of the population within that level that were current smokers, and that had mental illness either in the past 12 months, or in their lifetime but not in the past 12 months. Within each level we also calculated the proportion of the population who were current smokers by whether or not they had mental illness in the past 12 months, lifetime mental illness but not in the past 12 months, or did not meet criteria for lifetime mental illness. Tests of association were performed using the Rao-Scott adjustment for complex sample design [33].

We used logistic regression to assess the association between mental illness, each of the indicators of socioeconomic status, and current smoking status. Proportional hazards regression was used to assess the relationship between mental illness, each of the indicators of socioeconomic status, and time to smoking cessation, among ever smokers. We used age first started smoking as the start time for this analysis. For former smokers, age ceased smoking as used as the event time, while current smokers were censored at their current age. The complex survey design was accounted for using the SURVEYLOGISTIC and SURVEYPHREG procedures within SAS, with variances calculated using the jack-knife method of replicate weighting [32]. All analysis was conducted using Version 9.2 of SAS [34].

## Results

### Mental illness, indicators of socioeconomic disadvantage, and current smoking

For each socioeconomic indicator we calculated the proportion of the population that fell within each level of that indicator, and then within each level, the proportion of the population who smoked and the proportion who had mental disorder in the past 12 months (Table 1). Overall, the proportion of the adult population who were current smokers was 22.3% (95% CI: 20.9%-23.7%). The prevalence of 12-month mental disorder was estimated to be 20.0% (95% CI: 18.9%-21.1%), prevalence of lifetime disorder without 12-month disorder was 25.5% (95% CI: 24.1%-27.0%), prevalence of no lifetime disorder was 54.5% (53.1%-55.9%).

**Table 1:** Australian adults 16–85 years: Proportion of population, proportion who smoke and proportion with 12-month mental illness, by socioeconomic factors

**Parameter**	**Proportion of population**		**Current smokers**		**12-month mental illness**	
	**Percent**	**95% CI**	**Percent**	**95% CI**	**Percent**	**95% CI**
**Quintiles of household income—**						
Lowest quintile	16.8	15.7 - 17.8	24.4	22.0 - 26.8	23.6	20.9 - 26.3
Second quintile	17.0	15.7 - 18.3	23.1	19.5 - 26.7	20.0	17.0 - 23.1
Third quintile	17.0	15.8 - 18.2	21.9	18.5 - 25.3	21.3	18.0 - 24.5
Fourth quintile	16.8	15.7 - 18.0	22.2	19.5 - 25.0	17.7	14.4 - 20.9
Highest quintile	17.0	15.9 - 18.1	18.4	15.4 - 21.4	17.9	14.9 - 20.9
Not stated	15.4	14.2 - 16.6	23.7	19.3 - 28.2	19.3	15.8 - 22.8
			χ^2^ = 8.19, p = 0.15		χ^2^ = 23.2, p = 0.010	
**Quintiles of relative socioeconomic disadvantage—**						
Lowest quintile	16.7	14.6 - 18.8	29.6	26.1 - 33.1	21.4	18.4 - 24.4
Second quintile	18.5	16.1 - 20.9	26.8	23.8 - 29.9	20.9	18.5 - 23.2
Third quintile	20.0	17.7 - 22.3	21.8	19.1 - 24.5	21.3	18.0 - 24.7
Fourth quintile	21.8	19.1 - 24.5	19.8	17.2 - 22.3	21.2	18.7 - 23.7
Highest quintile	23.0	20.8 - 25.2	16.1	13.2 - 19.0	15.9	13.1 - 18.6
			χ^2^ = 53.5, p < 0.001		χ^2^ = 19.8, p = 0.011	
**Education—**						
Bachelor degree or higher	20.0	19.4 - 20.5	13.4	10.8 - 16.0	16.9	14.7 - 19.2
Diploma	8.5	8.1 - 8.9	21.4	16.6 - 26.2	21.9	17.9 - 25.8
Certificate I/II/III/IV	25.4	24.4 - 26.3	24.8	22.0 - 27.6	20.2	17.9 - 22.4
Year 12	14.4	13.4 - 15.5	22.2	18.8 - 25.6	22.4	19.2 - 25.5
Year 11	6.7	5.9 - 7.4	25.7	20.1 - 31.3	20.9	16.4 - 25.5
Year 10	13.6	12.6 - 14.6	26.8	23.0 - 30.5	21.9	17.7 - 26.0
Year 9 or below	11.4	10.6 - 12.2	25.5	22.2 - 28.9	17.6	14.4 - 20.7
			χ^2^ = 50.4, p < 0.001		χ^2^ = 24.4, p = 0.017	
**Whether ever been homeless—**						
Yes	3.0	2.6 - 3.5	61.0	53.3 - 68.8	53.6	45.7 - 61.5
No	97.0	96.5 - 97.4	21.1	19.7 - 22.4	18.9	17.9 - 20.0
			χ^2^ = 136.2, p < 0.001		χ^2^ = 253.5, p < 0.001	
**Whether ever in gaol, prison or correctional facility—**						
Yes	2.4	2.0 - 2.8	64.9	55.1 - 74.8	41.1	30.6 - 51.6
No	97.6	97.2 - 98.0	21.2	19.9 - 22.6	19.4	18.4 - 20.5
			χ^2^ = 99.3, p < 0.001		χ^2^ = 50.8, p < 0.001	
**Registered marital status—**						
Never married	32.5	31.3 - 33.7	30.9	28.6 - 33.2	27.9	25.7 - 30.0
Married	53.0	51.7 - 54.4	15.5	13.5 - 17.5	14.5	12.6 - 16.3
Widowed	4.5	4.2 - 4.9	13.3	10.0 - 16.6	16.7	12.0 - 21.4
Divorced	7.5	6.8 - 8.2	32.3	27.2 - 37.4	22.6	18.6 - 26.6
Separated	2.5	2.1 - 2.9	39.5	32.5 - 46.6	31.8	25.5 - 38.1
			χ^2^ = 193.5, p < 0.001		χ^2^ = 164.9, p < 0.001	
**Tenure type—**						
Owner without a mortgage	32.5	30.8 - 34.1	13.7	12.0 - 15.3	14.7	13.1 - 16.4
Owner with a mortgage	39.9	38.3 - 41.5	21.1	19.1 - 23.2	20.3	18.0 - 22.5
Renter	24.7	23.2 - 26.1	34.9	32.0 - 37.8	26.5	24.1 - 28.8
Other	2.9	2.5 - 3.4	26.2	19.0 - 33.5	19.1	12.6 - 25.6
			χ^2^ = 178.5, p < 0.001		χ^2^ = 96.3, p < 0.001	
**Financial difficulties—**						
Yes	14.5	13.4 - 15.6	39.8	35.3 - 44.2	39.1	35.3 - 42.8
No	85.5	84.4 - 86.6	19.3	18.0 - 20.7	16.7	15.5 - 17.9
			χ^2^ = 101.3, p < 0.001		χ^2^ = 172.9, p < 0.001	
**Labour force status—**						
Employed	65.2	64.8 - 65.6	23.4	21.7 - 25.1	20.3	18.7 - 21.8
Unemployed	2.6	2.5 - 2.7	50.8	38.9 - 62.6	29.4	20.3 - 38.4
Not in the labour force	32.2	31.8 - 32.5	17.6	15.6 - 19.7	18.6	16.9 - 20.3
			χ^2^ = 54.7, p < 0.001		χ^2^ = 23.9, p < 0.001	
**Occupation of main job—**						
Managers	8.9	8.0 - 9.9	17.6	14.4 - 20.8	18.7	14.9 - 22.4
Professionals	13.2	12.2 - 14.3	13.9	10.8 - 17.0	18.6	15.4 - 21.8
Technicians and Trades Workers	9.2	8.3 - 10.1	28.9	23.7 - 34.2	21.1	16.2 - 25.9
Community & Personal Services	6.0	5.4 - 6.7	24.9	19.8 - 30.0	22.5	17.0 - 28.1
Clerical and Administrative Workers	10.0	9.1 - 11.0	23.7	19.4 - 28.1	22.0	18.6 - 25.3
Sales Workers	6.4	5.6 - 7.2	20.7	15.3 - 26.1	20.8	16.2 - 25.4
Machinery Operators And Drivers	3.9	3.2 - 4.6	34.4	26.9 - 41.8	19.1	11.9 - 26.2
Labourers	7.1	6.3 - 7.9	36.6	30.8 - 42.5	20.6	15.2 - 25.9
Not applicable	35.3	34.9 - 35.7	20.1	18.0 - 22.1	19.3	17.7 - 21.0
			χ^2^ = 89.9, p < 0.001		χ^2^ = 32.6, p = 0.008	
**Family composition of household—**						
Couple with dependent children	33.4	32.1 - 34.7	17.6	15.0 - 20.2	18.7	16.1 - 21.3
One parent with dependent children	4.7	4.1 - 5.2	30.7	25.0 - 36.4	33.8	26.9 - 40.7
Couple only	27.3	26.5 - 28.0	16.7	15.1 - 18.3	14.4	13.0 - 15.9
Other one family households	14.5	13.1 - 15.9	33.0	28.4 - 37.6	23.5	18.8 - 28.2
Multiple family households	2.9	2.3 - 3.5	29.0	17.1 - 40.9	22.1	12.2 - 32.1
Lone person household	13.2	12.9 - 13.4	25.1	23.0 - 27.1	22.8	21.0 - 24.5
Group household	4.1	3.3 - 4.9	37.0	27.6 - 46.4	28.5	19.8 - 37.3
			χ^2^ = 95.9, p < 0.001		χ^2^ = 57.7, p < 0.001	
**Main source of income—**						
Employee cash income	55.5	54.5 - 56.6	23.6	21.6 - 25.5	20.5	19.0 - 22.0
Unincorporated business cash income	5.7	4.9 - 6.5	19.3	13.6 - 25.0	16.2	11.2 - 21.2
Government pensions and allowances	22.5	21.7 - 23.2	24.9	22.0 - 27.8	23.1	21.0 - 25.2
Other cash income	10.3	9.2 - 11.3	13.9	10.1 - 17.7	14.8	11.1 - 18.5
None of the above	6.0	5.3 - 6.7	17.5	12.4 - 22.6	15.7	10.8 - 20.6
			χ^2^ = 23.4, p < 0.001		χ^2^ = 44.6, p < 0.001	

**Figure 1:**
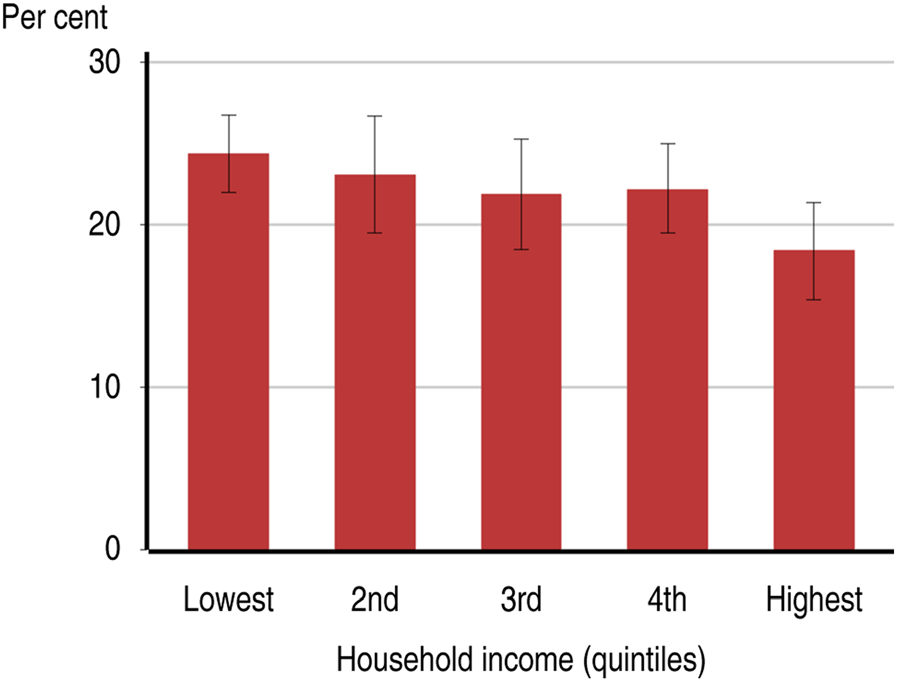
Proportion of Australian adults 16–85 years who smoke, by quintiles of family income.

Most of the indicators of socioeconomic disadvantage were associated with both current smoking status and mental illness. For instance, 24.4% of people in the lowest quintile of household income were current smokers (95% CI: 22.0%-26.8%) and 23.6% had mental illness in the past 12 months (95% CI: 20.9%-26.3%), compared with 18.4% who were current smokers (95% CI: 15.4%-21.4%) and 17.9% who had a 12-month mental illness (95% CI: 15.8%-22.8%) among people in the highest quintile of household income (Figure 1). All of the indicators of socioeconomic status were individually associated with current smoking (Table 1). All of these indicators were also associated with 12-month mental illness. While having ever been homeless or having ever been in a gaol, prison or correctional facility were both low prevalence indicators, each showed strong associations with current smoking and mental illness. Among the 3.0% of the adult population who had ever been homeless, 61.0% were current smokers (95% CI: 53.3%-68.8%), and 53.6% had 12-month mental illness (95% CI: 45.7%-61.5%). Among the 2.4% of the adult population who had ever been in gaol, prison or a correctional facility, 64.9% were current smokers (95% CI: 55.1%-74.8%), and 41.1% had a 12-month mental disorder (95% CI: 30.6%-51.6%).

### Combined association of mental illness and socioeconomic status

**Figure 2:**
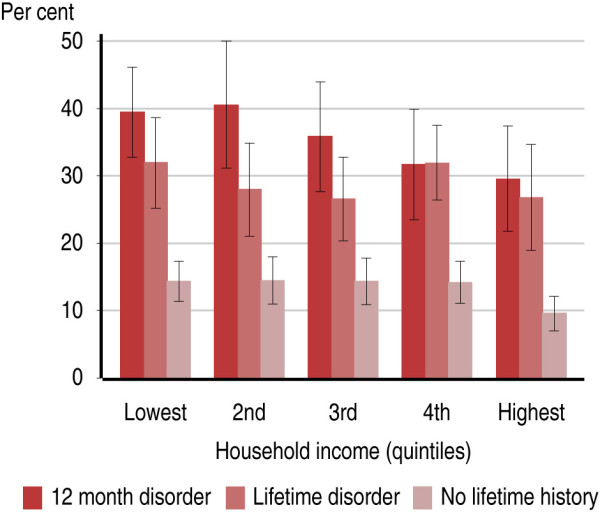
Proportion of Australian adults 16–85 years who smoke, by quintiles of family income and mental health status.

**Figure 3:**
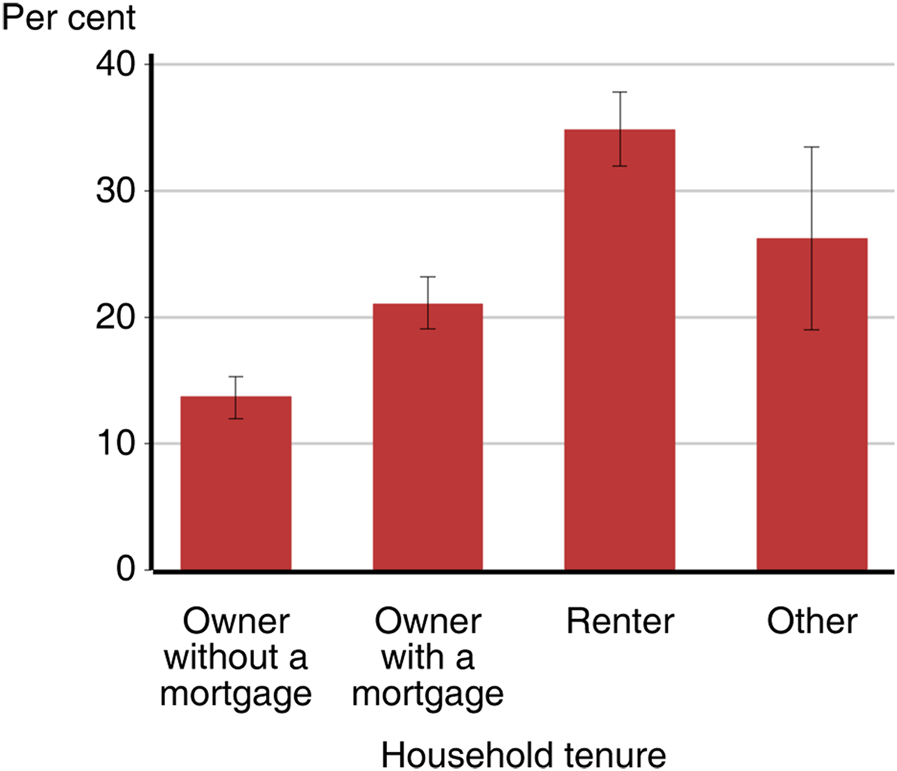
Proportion of Australian adults 16–85 years who smoke, by household tenure type.

**Figure 4:**
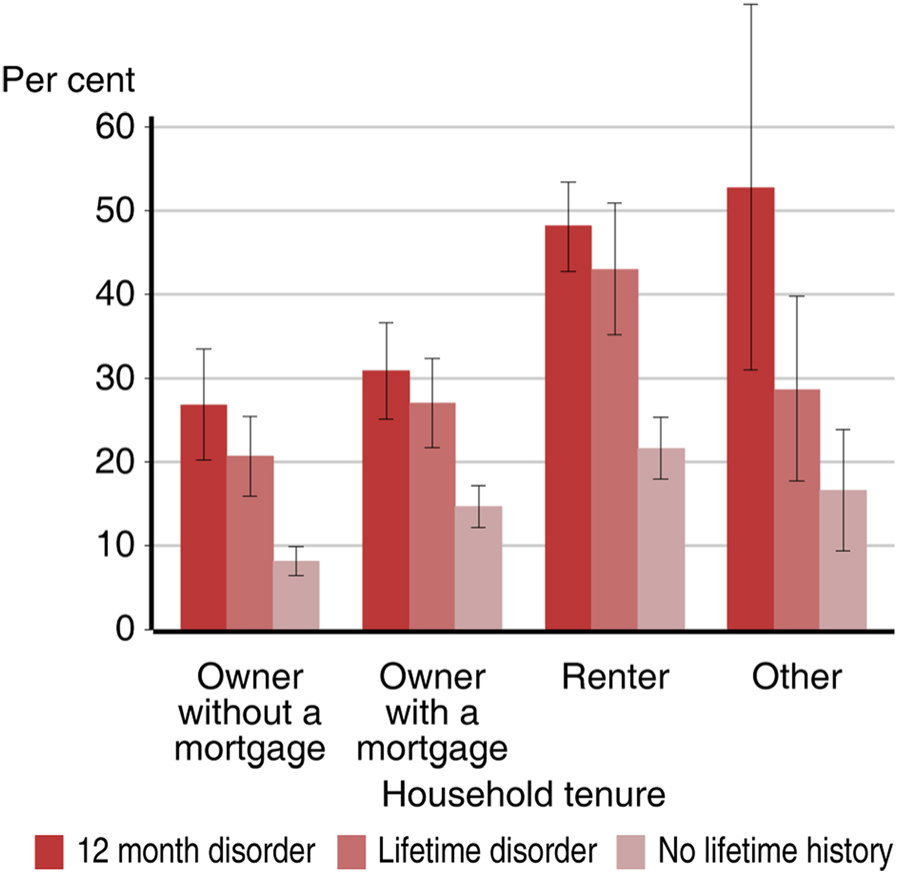
Proportion of Australian adults 16–85 years who smoke, by household tenure type.

Within each quintile of household income, there was a strong association between mental illness status and current smoking rates (Figure 2). For instance, in the lowest quintile of household income, 39.5% of those with 12-month mental illness were current smokers (95% CI: 32.8%-46.2%) while only 14.4% of those with no lifetime mental illness were current smokers (95% CI: 11.4%-17.3%). In the highest quintile of household income, 29.6% of those with 12-month mental illness were current smokers (95% CI: 21.8%-37.4%) while only 9.6% of those with no lifetime mental illness were current smokers (95% CI: 7.0%-12.1%). Similarly there is a large difference in smoking rates by household tenure type (Figure 3). An estimated 34.9% of adults living in rented accommodation were current smokers (95% CI: 32.0%-37.8%), while only 13.7% of people living in a home owned outright were current smokers (95% CI: 12.0%-15.3%). When split by mental illness status, this same gradient can be seen within those with and without mental illness, but there are significant differences in smoking rates by mental illness status for each household tenure type (Figure 4). Among people with 12-month mental illness, 48.2% of adults living in rented accommodation were current smokers (95% CI: 42.8%-53.5%) compared with 26.8% of adults living in a home owned outright (95% CI: 20.2%-33.5%). Among people with no lifetime mental disorder 21.6% of adults living in rented accommodation were current smokers (95% CI: 17.9%-25.4%) compared with 8.2% of adults living in a home owned outright (95% CI: 6.5%-9.9%). With the exception of household income, within each of these indicators of socioeconomic status, there remained a strong association between mental illness status and current smoking (Table 2).

**Table 2:** Australian adults 16–85 years: proportion who smoke by mental health status and socioeconomic factors

**Parameter**	**12 month mental illness**		**Lifetime mental illness**		**No mental illness**	
	**Percent**	**95% CI**	**Percent**	**95% CI**	**Percent**	**95% CI**
**Quintiles of household income—**						
Lowest quintile	39.5	32.8 - 46.2	32.0	25.2 - 38.7	14.4	11.4 - 17.3
Second quintile	40.6	31.2 - 50.0	28.0	21.0 - 34.9	14.5	11.0 - 18.0
Third quintile	35.9	27.7 - 44.0	26.6	20.4 - 32.8	14.4	10.9 - 17.8
Fourth quintile	31.7	23.5 - 39.9	31.9	26.4 - 37.5	14.2	11.1 - 17.3
Highest quintile	29.6	21.8 - 37.4	26.8	18.9 - 34.7	9.6	7.0 - 12.1
Not stated	38.2	29.1 - 47.3	35.4	20.4 - 50.5	14.7	10.4 - 19.0
	χ^2^ = 5.83, p = 0.320		χ^2^ = 3.60, p = 0.612		χ^2^ = 7.09, p = 0.218	
**Quintiles of relative socioeconomic disadvantage—**						
Lowest quintile	44.9	36.4 - 53.4	38.1	30.1 - 46.0	18.9	14.9 - 22.9
Second quintile	45.1	37.3 - 53.0	32.2	26.3 - 38.2	16.5	13.1 - 20.0
Third quintile	36.7	28.7 - 44.7	29.2	21.6 - 36.8	12.1	9.1 - 15.2
Fourth quintile	29.4	22.8 - 36.1	25.1	19.5 - 30.7	13.5	10.1 - 17.0
Highest quintile	26.0	18.0 - 34.0	26.1	17.8 - 34.3	9.6	7.2 - 12.0
	χ^2^ = 19.4, p < 0.001		χ^2^ = 8.58, p = 0.072		χ^2^ = 20.4, p < 0.001	
**Education—**						
Bachelor degree or higher	23.5	16.7 - 30.3	18.4	11.8 - 25.0	7.7	5.4 - 10.1
Diploma	38.5	26.3 - 50.7	25.2	12.1 - 38.3	12.9	7.9 - 17.8
Certificate I/II/III/IV	39.4	32.7 - 46.0	32.7	27.4 - 38.1	14.7	11.2 - 18.2
Year 12	29.0	21.8 - 36.2	31.7	19.4 - 44.0	15.9	11.5 - 20.3
Year 11	44.0	29.6 - 58.3	29.8	17.1 - 42.4	16.7	9.5 - 24.0
Year 10	41.8	31.5 - 52.1	39.7	30.2 - 49.1	15.2	11.3 - 19.0
Year 9 or below	45.0	33.4 - 56.7	35.9	26.6 - 45.1	15.7	12.1 - 19.3
	χ^2^ = 19.0, p = 0.004		χ^2^ = 17.4, p = 0.008		χ^2^ = 17.2, p = 0.008	
**Whether ever been homeless—**						
Yes	63.6	51.6 - 75.7	67.7	58.1 - 77.3	31.5	12.5 - 50.5
No	33.7	30.1 - 37.4	28.2	24.8 - 31.6	13.5	12.1 - 15.0
	χ^2^ = 23.6, p < 0.001		χ^2^ = 61.8, p < 0.001		χ^2^ = 7.2, p = 0.007	
**Whether ever in gaol, prison or correctional facility—**						
Yes	80.0	70.1 - 89.9	63.3	46.6 - 80.1	35.5	5.9 - 65.0
No	33.9	30.4 - 37.4	28.5	25.1 - 32.0	13.5	12.1 - 14.9
	χ^2^ = 67.5, p < 0.001		χ^2^ = 19.0, p < 0.001		χ^2^ = 5.75, p = 0.016	
**Registered marital status—**						
Never married	46.3	41.4 - 51.3	40.5	33.9 - 47.1	17.4	15.1 - 19.8
Married	25.0	19.2 - 30.8	22.2	17.4 - 27.0	10.3	8.6 - 12.0
Widowed	16.1	3.5 - 28.8	19.0	9.2 - 28.9	10.8	8.2 - 13.5
Divorced	37.5	28.2 - 46.8	40.1	30.3 - 49.8	23.6	16.1 - 31.0
Separated	44.4	35.2 - 53.6	38.0	22.5 - 53.5	36.6	24.4 - 48.8
	χ^2^ = 55.3, p < 0.001		χ^2^ = 39.7, p < 0.001		χ^2^ = 79.7, p < 0.001	
**Tenure type—**						
Owner without a mortgage	26.8	20.2 - 33.5	20.7	15.9 - 25.5	8.2	6.5 - 9.9
Owner with a mortgage	30.9	25.1 - 36.6	27.0	21.7 - 32.4	14.6	12.1 - 17.1
Renter	48.2	42.8 - 53.5	43.0	35.2 - 50.9	21.6	17.9 - 25.4
Other	52.8	31.0 - 74.6	28.7	17.7 - 39.8	16.6	9.4 - 23.9
	χ^2^ = 31.3, p < 0.001		χ^2^ = 33.7, p < 0.001		χ^2^ = 55.7, p < 0.001	
**Financial difficulties—**						
Yes	50.1	43.7 - 56.6	41.9	35.2 - 48.7	25.8	18.1 - 33.5
No	30.7	26.6 - 34.7	27.6	23.8 - 31.4	12.5	11.2 - 13.8
	χ^2^ = 27.9 p < 0.001		χ^2^ = 14.4, p < 0.001		χ^2^ = 20.9, p < 0.001	
**Labour force status—**						
Employed	36.0	31.6 - 40.5	30.4	26.9 - 34.0	14.9	12.8 - 16.9
Unemployed	57.8	40.2 - 75.3	74.3	44.8 - 100.0	31.1	18.8 - 43.4
Not in the labour force	33.7	26.9 - 40.5	23.7	19.4 - 27.9	10.5	8.8 - 12.1
	χ^2^ = 6.21, p = 0.045		χ^2^ = 23.2, p < 0.001		χ^2^ = 27.6, p < 0.001	
**Occupation of main job—**						
Managers	26.3	16.3 - 36.4	20.4	14.7 - 26.1	12.9	8.6 - 17.1
Professionals	21.9	13.6 - 30.2	21.0	12.3 - 29.7	7.6	5.2 - 10.1
Technicians and Trades Workers	44.7	34.8 - 54.5	36.9	26.4 - 47.4	17.5	11.8 - 23.3
Community and Personal Services	39.3	26.4 - 52.3	21.8	13.0 - 30.7	20.2	12.9 - 27.5
Clerical and Administrative Workers	33.3	22.9 - 43.8	35.3	23.5 - 47.1	13.2	8.8 - 17.6
Sales Workers	38.4	25.1 - 51.7	19.8	9.5 - 30.0	14.9	7.9 - 21.9
Machinery Operators And Drivers	39.0	17.4 - 60.6	41.5	27.9 - 55.2	27.2	17.3 - 37.2
Labourers	55.9	45.3 - 66.4	53.9	41.4 - 66.3	20.1	11.7 - 28.5
Not applicable	36.7	30.5 - 42.8	28.0	22.5 - 33.5	11.6	9.8 - 13.4
	χ^2^ = 26.7, p < 0.001		χ^2^ = 42.1, p < 0.001		χ^2^ = 36.5, p < 0.001	
**Family composition of household—**						
Couple with dependent children	32.9	25.9 - 40.0	23.5	18.3 - 28.7	10.2	7.7 - 12.7
One parent with dependent children	36.0	25.4 - 46.6	32.4	20.4 - 44.4	25.0	16.2 - 33.9
Couple only	27.9	21.7 - 34.2	24.2	20.8 - 27.6	10.3	8.7 - 12.0
Other one family households	44.8	33.6 - 56.1	44.8	33.6 - 56.0	21.7	16.1 - 27.3
Multiple family households	38.4	15.5 - 61.2	40.5	1.6 - 79.3	20.3	9.1 - 31.5
Lone person household	38.2	34.2 - 42.3	33.8	29.2 - 38.5	15.1	13.0 - 17.1
Group household	49.9	34.2 - 65.6	39.6	13.1 - 66.1	25.3	14.6 - 36.0
	χ^2^ = 14.3, p = 0.030		χ^2^ = 20.3, p = 0.002		χ^2^ = 55.0, p < 0.001	
**Main source of income—**						
Employee cash income	35.7	31.3 - 40.2	31.3	26.9 - 35.7	15.0	12.8 - 17.3
Unincorporated business cash income	31.9	16.3 - 47.6	23.1	9.9 - 36.4	12.1	7.7 - 16.5
Government pensions & allowances	43.4	36.9 - 49.9	32.9	25.6 - 40.2	13.1	10.5 - 15.6
Other cash income	25.8	11.4 - 40.2	19.7	10.6 - 28.9	9.0	5.7 - 12.4
None of the above	22.1	8.8 - 35.4	28.4	9.6 - 47.1	13.4	7.8 - 19.1
	χ^2^ = 10.7, p = 0.030		χ^2^ = 5.39, p = 0.250		χ^2^ = 8.4, p = 0.079	

### Factors associated with current smoking

We fitted a series of univariate logistic regression models with current smoking as the outcome measure to estimate the association with mental illness or socioeconomic status. Each of these models also adjusted for the age and sex of the respondent. After adjusting for age and sex, each of the indicators was significantly associated with current smoking status. Strongest univariate associations were observed for having ever been homeless (OR 5.45; 95% CI: 3.91-7.60), and whether ever been in gaol, prison or a correctional facility (OR 6.54; 95% CI: 4.10-10.30). These are relatively low prevalence factors. Most of the higher prevalence risk factors showed more modest associations, with the strongest of these being for the 20.0% of the adult population with 12-month mental illness (OR: 3.26; 95% CI:2.69-3.95) (Table 3).

**Table 3:** Australian adults 16–18 years: Univariate and multivariate odds ratios for smoking, associated with socioeconomic factors and mental illness

**Parameter**	**Univariate odds ratios (a)**		**Multivariate odds ratios (b)**	
	**Odds ratio**	**95% CI**	**Odds ratio**	**95% CI**
**Mental health status—**				
12 month disorder	3.26	2.69 - 3.95	2.43	1.97 - 3.01
Lifetime disorder	2.52	2.04 - 3.12	2.15	1.72 - 2.70
No mental illness	1.00		1.00	
**Quintiles of household income—**				
Lowest quintile	2.29	1.76 - 3.00	(c)	
Second quintile	1.64	1.22 - 2.21		
Third quintile	1.36	1.02 - 1.81		
Fourth quintile	1.28	0.99 - 1.65		
Highest quintile	1.00			
Not stated	1.56	1.14 - 2.13		
**Quintiles of relative socioeconomic disadvantage—**				
Lowest quintile	2.26	1.72 - 2.96	1.23	0.92 - 1.63
Second quintile	2.01	1.51 - 2.67	1.27	0.97 - 1.68
Third quintile	1.46	1.17 - 1.83	1.05	0.81 - 1.35
Fourth quintile	1.25	0.96 - 1.63	0.97	0.76 - 1.23
Highest quintile	1.00		1.00	
**Education—**				
Bachelor degree or higher	0.35	0.26 - 0.48	0.48	0.34 - 0.68
Diploma	0.68	0.47 - 0.98	0.81	0.56 - 1.17
Certificate I/II/III/IV	0.80	0.63 - 1.02	0.83	0.63 - 1.09
Year 12	0.62	0.48 - 0.82	0.72	0.54 - 0.95
Year 11	0.81	0.56 - 1.16	0.94	0.63 - 1.40
Year 10	1.00		1.00	
Year 9 or below	1.44	1.12 - 1.85	1.28	0.98 - 1.67
**Whether ever been homeless—**				
Yes	5.45	3.91 - 7.60	1.82	1.23 - 2.69
No	1.00		1.00	
**Whether ever in gaol, prison or correctional facility—**				
Yes	6.54	4.10 - 10.30	2.64	1.44 - 4.87
No	1.00		1.00	
**Registered marital status—**				
Never married	2.49	2.00 - 3.10	1.60	1.25 - 2.06
Married	1.00		1.00	
Widowed	1.52	1.04 - 2.24	1.32	0.85 - 2.05
Divorced	2.70	2.07 - 3.51	1.81	1.29 - 2.53
Separated	3.48	2.49 - 4.86	2.09	1.35 - 3.22
**Tenure type—**				
Owner without a mortgage	1.00		1.00	
Owner with a mortgage	1.27	1.04 - 1.54	1.32	1.08 - 1.61
Renter	2.60	2.10 - 3.21	1.96	1.52 - 2.52
Other	1.85	1.17 - 2.92	1.78	1.15 - 2.75
**Financial difficulties—**				
Yes	2.51	2.05 - 3.06	1.41	1.11 - 1.80
No	1.00		1.00	
**Labour force status—**				
Employed	1.00		1.00	
Unemployed	3.27	2.07 - 5.17	2.25	1.12 - 4.54
Not in the labour force	1.10	0.88 - 1.37	0.93	0.42 - 2.05
**Occupation of main job—**				
Managers	1.00		1.00	
Professionals	0.77	0.55 - 1.08	0.79	0.55 - 1.16
Technicians and Trades Workers	1.72	1.20 - 2.45	1.24	0.88 - 1.74
Community and Personal Service Workers	1.67	1.19 - 2.35	1.07	0.73 - 1.58
Clerical and Administrative Workers	1.57	1.12 - 2.21	1.18	0.85 - 1.64
Sales Workers	1.28	0.83 - 1.98	1.00	0.65 - 1.54
Machinery Operators And Drivers	2.32	1.46 - 3.68	1.32	0.81 - 2.14
Labourers	2.72	1.99 - 3.73	1.70	1.25 - 2.30
Not applicable	1.84	1.38 - 2.44	1.01	0.42 - 2.42
**Family composition of household—**				
Couple family with dependent children	0.65	0.53 - 0.81	0.70	0.57 - 0.87
One parent family with dependent children	1.53	1.16 - 2.03	0.66	0.46 - 0.94
Couple only	1.00		1.00	
Other one family households	1.92	1.49 - 2.46	1.65	1.24 - 2.18
Multiple family households	1.63	0.91 - 2.92	1.50	0.76 - 2.96
Lone person household	1.64	1.41 - 1.91	0.87	0.69 - 1.09
Group household	1.81	1.11 - 2.94	1.13	0.67 - 1.90
**Main source of income—**				
Employee cash income	1.00		(c)	
Unincorporated business cash income	0.82	0.55 - 1.21		
Government cash pensions and allowances	1.91	1.51 - 2.41		
Other cash income	0.98	0.65 - 1.46		
None of the above	0.73	0.50 - 1.07		

(a) Univariate odds ratios adjusting for age and sex.

(b) Multivariate odds ratios adjusting for all variables shown and age and sex.

(c) Not significantly associated with smoking status in multivariate model and eliminated from final model**.**

We also fitted a multivariate model considering the indicators simultaneously. We eliminated non-significant variables to develop a most parsimonious model, as some of the indicators of socioeconomic status were highly correlated. Household income, main source of cash income and relative socioeconomic disadvantage were highly correlated. Relative socioeconomic disadvantage was retained in the model as the most strongly associated of the three once the other indicators were included in the model. In the full model before these variables were eliminated from the model, household income (p = 0.961), and main source of household income (p = 0.506) were not significantly associated with smoking status. The univariate associations were all attenuated in the multivariate model reflecting the associations between the various indicators. The strongest risk factors for current smoking were ever being in gaol, prison or correctional facility (OR 2.64; 95% CI: 1.44-4.87; 2.4% of the population), having a mental illness in the past 12 months (OR 2.43; 95% CI: 1.97-3.01; 20.0% of the population), or having lifetime mental illness but not in the past 12 months (OR 2.15; 95% CI: 1.72-2.70; 25.5% of the population), being unemployed (OR 2.25; 95% CI: 1.12-4.54; 2.6% of the population), being separated (OR 2.09; 95% CI: 1.35-3.22; 2.5% of the population), and living in rented accommodation (OR 1.96; 95% CI: 1.52-2.52, 24.7% of the population).

### Factors associated with smoking cessation

We fitted a series of proportional hazards regression models for ever smokers with time to smoking cessation as the outcome measure to estimate the association with mental illness or socioeconomic status. Each of these models also adjusted for the age and sex of the respondent. After adjusting for age and sex, each of the indicators was significantly associated with time to quit smoking. Comparably sized associations with smoking cessation were seen for most of the indicators (Table 4). Those with a 12-month mental disorder were less likely to quit smoking (HR 0.64; 95% CI: 0.55-0.75), as were those living in the lowest quintile of relative socioeconomic disadvantage (HR: 0.55; 95% CI: 0.44-0.70), and those in the lowest quintile of household income (HR: 0.53; 95% CI: 0.42-0.66). Several of the indicators were eliminated in the final multivariate model including household income (p = 0.533), whether ever homeless (p = 0.749), family composition (p = 0.433), main source of income (p = 0.638) and labour force status (p = 0.929). The strongest hazards for not quitting smoking were associated with renters compared with those living in homes owned outright (HR: 0.64; 95% CI: 0.52-0.78), labourers compared with managers (HR: 0.65; 95% CI: 0.46-0.92), and 12-month mental illness (HR: 0.77; 95% CI: 0.65-0.91) or lifetime mental illness (HR: 0.77; 95% CI: 0.64-0.92).

**Table 4:** Hazard ratios for smoking cessation, by mental health status and socioeconomic factors

**Parameter**	**Univariate hazard ratios (a)**		**Multivariate hazard ratios (b)**	
	**Hazard ratio**	**95% CI**	**Hazard ratio**	**95% CI**
**Mental health status—**				
12 month disorder	0.64	0.55 - 0.75	0.77	0.65 - 0.91
Lifetime disorder	0.72	0.61 - 0.84	0.77	0.64 - 0.92
No mental illness	1.00		1.00	
**Quintiles of household income—**				
Lowest quintile	0.53	0.42 - 0.66	(c)	
Second quintile	0.64	0.51 - 0.81		
Third quintile	0.74	0.59 - 0.94		
Fourth quintile	0.72	0.58 - 0.89		
Highest quintile	1.00			
Not stated	0.58	0.46 - 0.73		
**Quintiles of relative socioeconomic disadvantage—**				
Lowest quintile	0.55	0.44 - 0.70	0.80	0.63 - 1.02
Second quintile	0.61	0.50 - 0.74	0.80	0.65 - 0.98
Third quintile	0.71	0.59 - 0.85	0.84	0.69 - 1.02
Fourth quintile	0.74	0.60 - 0.91	0.85	0.69 - 1.05
Highest quintile	1.00		1.00	
**Education—**				
Bachelor degree or higher	1.66	1.28 - 2.16	1.31	0.98 - 1.73
Diploma	1.81	1.37 - 2.41	1.50	1.13 - 1.98
Certificate I/II/III/IV	1.17	0.97 - 1.42	1.07	0.87 - 1.33
Year 12	1.37	1.11 - 1.69	1.19	0.95 - 1.51
Year 11	1.23	0.86 - 1.76	1.14	0.78 - 1.67
Year 10	1.00		1.00	
Year 9 or below	0.79	0.65 - 0.96	0.80	0.64 - 0.99
**Whether ever been homeless—**				
Yes	0.49	0.37 - 0.65	(c)	
No	1.00			
**Whether ever in gaol, prison or correctional facility—**				
Yes	0.49	0.35 - 0.68	0.70	0.49 - 1.01
No	1.00		1.00	
**Registered marital status—**				
Never married	0.62	0.51 - 0.75	0.76	0.62 - 0.93
Married	1.00		1.00	
Widowed	0.64	0.53 - 0.78	0.73	0.59 - 0.90
Divorced	0.65	0.53 - 0.80	0.77	0.62 - 0.96
Separated	0.58	0.45 - 0.73	0.78	0.59 - 1.02
**Tenure type—**				
Owner without a mortgage	1.00		1.00	
Owner with a mortgage	0.90	0.77 - 1.05	0.84	0.70 - 1.00
Renter	0.54	0.45 - 0.65	0.64	0.52 - 0.78
Other	0.58	0.43 - 0.79	0.57	0.42 - 0.78
**Financial difficulties—**				
Yes	0.63	0.51 - 0.77	0.84	0.66 - 1.08
No	1.00		1.00	
**Labour force status—**				
Employed	1.00		(c)	
Unemployed	0.60	0.38 - 0.95		
Not in the labour force	0.81	0.69 - 0.94		
**Occupation of main job—**				
Managers	1.00		1.00	
Professionals	1.22	0.87 - 1.70	1.15	0.82 - 1.61
Technicians and Trades Workers	0.75	0.57 - 0.98	0.89	0.68 - 1.16
Community and Personal Service Workers	0.70	0.49 - 0.99	0.76	0.52 - 1.12
Clerical and Administrative Workers	0.86	0.62 - 1.20	0.85	0.58 - 1.23
Sales Workers	0.98	0.67 - 1.45	1.11	0.75 - 1.65
Machinery Operators And Drivers	0.62	0.45 - 0.87	0.74	0.50 - 1.11
Labourers	0.52	0.38 - 0.71	0.65	0.46 - 0.92
Not applicable	0.65	0.53 - 0.81	0.83	0.64 - 1.07
**Family composition of household—**				
Couple family with dependent children	1.28	1.09 - 1.51	(c)	
One parent family with dependent children	0.85	0.56 - 1.30		
Couple only	1.00			
Other one family households	0.69	0.55 - 0.86		
Multiple family households	0.76	0.42 - 1.40		
Lone person household	0.67	0.60 - 0.75		
Group household	0.73	0.50 - 1.06		
**Main source of income—**				
Employee cash income	1.00		(c)	
Unincorporated business cash income	1.05	0.80 - 1.39		
Government cash pensions and allowance	0.69	0.58 - 0.82		
Other cash income	1.02	0.80 - 1.30		
None of the above	0.89	0.70 - 1.13		

(a) Univariate hazard ratios adjusting for age and sex.

(b) Multivariate hazard ratios adjusting for all variables shown and age and sex.

(c) Not significantly associated with smoking cessation in multivariate model and eliminated from final model.

## Discussion

There are substantial socioeconomic gradients in rates of both current smoking and smoking cessation among adults, with people in more disadvantaged groups both more likely to be current smokers, and have lower quit rates. When considered in multivariate models, independent associations were found between a range of markers of disadvantage and smoking status. In terms of current smoking, our results on socioeconomic indicators are consistent with prior research, although psychosocial variables such as mental health status typically have not been included in this research previously [35-38].

Our findings do not support our original null hypothesis that there would be no independent association between mental health status and smoking after adjusting for a comprehensive set of markers of socioeconomic disadvantage. Indeed mental illness was the most prevalent risk factor with a strong independent association with current smoking and reduced likelihood of smoking cessation. This supports previous findings indicating the high burden of smoking associated with mental disorders such as depression and anxiety [4-6], which may be the most prevalent risk factor strongly associated with smoking behaviours. These results indicate that previous findings were not merely presenting a confounded relationship with socioeconomic status. While these data do not address the question of whether this association is causal and if so, in what direction, they do raise the question as to whether efforts to control tobacco use and to promote smoking cessation in this group would be more effective if the impacts of mental illness were explicitly incorporated into their design.

Similarly the reverse hypothesis, that there would be no independent association between socioeconomic status and smoking after adjusting for mental health status is not supported by these data. Even in those with no lifetime mental illness, gradients in both current smoking status and smoking cessation can be observed for several indicators of socioeconomic status. Living in rented accommodation remaining strongly associated with both current smoking and smoking cessation in the multivariate models, and being unemployed or ever having been in gaol, prison or a correctional facility strongly associated with current smoking.

There are several possible mechanisms that may explain an independent association between smoking and common mental disorders [5]. Mental illness may be a factor in smoking initiation or addiction to nicotine. Smoking may be a risk factor for onset of mental illness. Alternatively both smoking and mental illness could be linked to common genetic, biological or environmental factors. There is some evidence to support each of these possible mechanisms [39]. Depression and anxiety in teenagers have been found to be strong predictors of smoking experimentation and the transition to daily smoking [40-42]. Smoking has also been associated with the onset of psychiatric symptoms in teenagers [42,43]. While it is possible that separate causal mechanisms may operate in both directions, other studies have identified common risk factors to both smoking and mental illness [44-47].

There are well-established biological mechanisms that help explain why smoking may be linked to mental illness. Nicotine is a psychostimulant that affects several neuroregulators that influence both mood and behaviour [48,49]. Early reports suggested that nicotine cessation can also precipitate depressive symptoms, particularly in people with a history of major depression [50,51]. Nicotine administration can relieve symptoms of both depression and anxiety [52-54]. Levels of cortisol, a component of the hypothalamic-pituitary-adrenal axis system that responds to stress, can be affected by nicotine [39,55]. More recent studies have suggested that nicotine withdrawal symptoms can be quite similar to symptoms of anxiety and depression which may further reinforce smoking behaviours in people with these disorders [54,56]. The knowledge that people with mental illness may perceive there to be some therapeutic benefit from nicotine led to the self-medication hypothesis, which suggests that people with mental illness may choose to smoke as it is a simple and readily accessible means to control symptoms of mental illness [57]. However, levels of anxiety and depression in ex-smokers after the withdrawal period may be lower than immediately after smoking in current smokers suggesting that perceived benefit of smoking for people with depression and anxiety may merely reflect the similarity between nicotine withdrawal symptoms and symptoms of anxiety or depression [39,58].

The finding that smoking rates are higher among disadvantaged groups is not new. However, to the best of our knowledge, the finding that strong gradients in smoking, and smoking cessation, are observed by mental illness status within both disadvantaged and non-disadvantaged groups has not previously been reported. We also found that smoking cessation rates are lower in socioeconomically disadvantaged groups and in those with common mental disorders, and that significantly lower cessation rates are observed in disadvantaged groups after controlling for mental illness. This is a more controversial finding.

Other reports have suggested that while smoking initiation rates may be higher in disadvantaged groups, smoking cessation rates are equal in all groups, although these reports tend to rely on coarse measures, and flawed interpretations of broad population measures. Two measures commonly cited to suggest that quit rates are equal across demographic groups are the proportion of population groups who are ex-smokers [19,59-61], and the absolute percentage point reduction in smoking rates in various groups [19,62]. For example, Australia’s National Preventative Health Taskforce reported that:

“Most disparities in smoking rates between socioeconomic groups in Australia result from differences in uptake rather than in cessation…around 30% of people can be classified as ex-smokers, regardless of the level of neighbourhood disadvantage.” [19, p. 12]

However, the proportion of ex-smokers is not a reliable estimate of cessation rate as the denominator is all persons, not smokers. Consider the following hypothetical example. Suppose two groups of 100 smokers are followed for a period of time, and 20 people quit smoking in each group. However group A comes from a relatively disadvantaged group with a total population of 200 people while the less disadvantaged group B has a total population of 400 people. In group A 20 of 200 people, or 10% are ex-smokers, while only 5% of group B are ex-smokers, despite both groups having the same smoking cessation rate. Indeed, if only 10 people had quit smoking in group A, both groups would have shown the same proportion of ex-smokers despite the smoking cessation rate being half that in group A compared with group B. As smoking rates are substantially higher in areas of high neighbourhood disadvantage, the data presented by Australia’s National Preventative Health Taskforce are consistent with our findings, as equivalent proportions of ex-smokers imply lower smoking cessation in groups with higher smoking initiation rates. However, these data have not previously been interpreted in this way. In a similar vein, the National Preventative Health Taskforce also reported that smoking rates had declined between 1989–90 and 2004–5 from 33% to 29% in areas in the highest quintile of socioeconomic disadvantage, and from 23% to 17% in areas in the lowest quintile of socioeconomic disadvantage [19]. The argument that graphs of time trends in smoking rates in different groups are represented by approximately parallel trend lines has been advanced to suggest that cessation rates are equal in different groups [19,59,62]. However, again this does not account for the differences in smoking prevalence in the groups. If cessation rates were equal in different groups, trend lines in smoking prevalence, if linear, would be expected to converge to the same point, not be parallel. Considering again our hypothetical example, with the same smoking cessation rate in each group the smoking rate declines by 10 percentage points, from 50% to 40% in group A, and by 5 percentage points, from 25% to 20% in group B. If group A had only half the smoking cessation rate of group B, the decline in smoking rate would have been 5 percentage points from 50% to 45%.

The results of our study support the general principle of developing tobacco control strategies that specifically address the needs of disadvantaged groups with high smoking rates. There were substantial gradients in smoking rates observed across many of the indicators included in this study. These reflect not only the high relative burden of smoking associated with various forms of disadvantage, but the extent to which broad-based tobacco control efforts have had their greatest success among those who have fewer additional disadvantages in their lives. The strongly skewed sociodemographic of current smokers now has emerged from a much more homogeneous population of smokers thirty or forty years ago, when smoking was actually more common among the more affluent and well-educated [63-65].

For affluent adults with no history of mental illness, current smoking rates are now below 10% while rates are over 40% for those with 12-month mental illness and one of several indicators of socioeconomic disadvantage.

There are a number of programmes that have been developed that address smoking within mental health treatment settings, such as the Tobacco and Mental Illness project in South Australia [66], and Mental Health Tobacco Recovery in New Jersey [67]. While these programmes include components to assist with the transition to community-based living, including the use of peer support workers [68], they focus on people with serious mental illness and generally recruit from specialist psychiatric services.

A number of strategies have been proposed as to how to address the high rate of smoking among disadvantaged groups and people with common mental illnesses [20,57,69]. Broadly these can be considered as either programmes that work with individuals or in small groups to support smoking cessation, and approaches that modify broad population health interventions to more specifically target disadvantaged groups. An intermediary approach is exemplified by the *Tackling Tobacco* programme initiated by the New South Wales Cancer Council. This programme is based on the likelihood that many people from disadvantaged backgrounds are in contact with health or social services for other reasons as a result of that disadvantage. This may then be a way of targeting this group. The programme seeks to integrate tobacco control into the services provided by a range of social and community sector organisations [70]. As such it seeks to denormalise smoking in sectors working with disadvantaged people where smoking behaviours are commonplace, and to increase the impact of brief interventions and support for cessation beyond the health sector. So far this programme has been piloted and qualitatively reviewed [71,72] and a randomised controlled trial is underway to investigate the effectiveness of this type of approach in achieving smoking cessation within disadvantaged groups [73]. Preliminary data suggest that both the organisations and their clients are enthusiastic to have smoking cessation activities provided through these services [71,72,74].

Another possible approach to addressing smoking among disadvantaged groups is to adapt population health-based methods. The principal components of population health-based smoking cessation efforts, such as controlling supply, restricting all forms of promotion, increasing price, advertising health consequences and educating young people about them, denormalising or stigmatising smoking, and restricting use in public places, have generally not been tailored for people from specific demographic groups.

The design of population health interventions such as education and denormalisation can be considered within the framework of social marketing [75]. Although targeted approaches, or market segmentation, are widely used in the marketing of commercial products and services, including historically in the promotion of cigarettes [76-78], this approach has not been strongly embraced in population health. Bloom and Novelli note that treating certain groups with special attention “is not consistent with the egalitarian and antidiscriminatory philosophies that pervade many social agencies” and that during program planning and implementation there is “a constant problem about whether to divide limited resources or simply take a general audience route” [79]. This argument has also been made specifically in the area of tobacco control. Indeed, it has been suggested that money spent on targeted anti-smoking efforts is money wasted as it is money taken away from the most successful broad population-based approaches [80]. Alternatively it has been argued that developing both targeted and broad population-based strategies would undermine both as no strategy would then be adequately funded to a level that could have impact [81]. These arguments rest on the assumption that all population subgroups benefit equally from broad reach interventions, which is counter to the increasing burden of smoking concentrated in disadvantaged groups.

Of the various broad-based population-health intervention approaches to smoking cessation, most have greater impact in more advantaged groups [82]. Only price increases through taxation have been suggested to be more effective in disadvantaged groups [82,83]. However, the data on the effectiveness of taxation increases in reducing smoking in disadvantaged groups is equivocal, and others have questioned this finding [84,85]. The question of whether any one aspect of tobacco control is more or less successful in disadvantaged groups misses the fact that the successes of tobacco control overall have come from the implementation of programs that employ multiple strategies.

While the preference for broad-based programmes may be based on egalitarian principles [79], in fact this focus on broad-reach interventions may promote inequality if these interventions are most successful amongst advantaged subgroups. Two common themes in health promotion in tobacco control are the long-term health consequences of smoking and establishing smoking as a stigmatised behaviour. The promotion of long-term health consequences may be less motivating of behaviour change in people whose ability to project in the longer term is limited by pressing life circumstances or stress or whose cognitive skills are impaired by psychological distress [86,87]. Similarly the impact of the stigmatisation of smoking behaviours may be less motivating in people who are also stigmatised by other forms of disadvantage [88]. Ceci and Papierno have argued that reliance on universal strategies will always result in the widening of gaps between advantaged and disadvantaged groups as the resources, skills and opportunities of advantaged groups act to increase their chances of utilising and benefitting from any universal strategy [89]. Niederdeppe and colleagues point out that mass media campaigns, in particular, are often differentially effective in advantaged groups for multiple reasons: levels of exposure, levels of persuasiveness, opportunities to change, and access to supports all vary by level of disadvantage [90,91].

Marsh and McKay noted that while price increases have played an important part in overall tobacco control, perversely some of the heaviest smokers are also among the poorest and pay a high financial price for their smoking. Yet their levels of addiction, financial literacy and overall decision making result in this strategy yielding lower results among the poorest smokers [92].

There is likely to be an important role for both types of approach in addressing the issue of smoking and disadvantage. Initiatives based around use of services can reach groups where broad-based strategies have little or no penetration, and these services are often the best way of reaching people with the most severe and multiple disadvantages. At the same time, not all disadvantaged smokers are in contact with services, and service-based programmes will only reach a proportion of the target audience. For example, the majority of people with mental disorders such as anxiety or depression are not in contact with services for these problems, although there is no difference in smoking or smoking cessation rates between those who do and do not use services [6,21]. In responding to the association between markers of disadvantage and smoking, the strategies recommended by Australia’s National Preventative Health Taskforce have aimed to target the small proportion of people with very high levels of disadvantage, such as people who are currently homeless or in a correctional facility or long-term residents of psychiatric facilities [19]. While there is no doubt that smoking rates are very high in these groups, our data show that the gradients in smoking status extend to a significantly larger proportion of the population. This has implications for tobacco control efforts. While it is possible to develop programs within institutional settings or that use street workers to target homeless people, targeted population-based strategies will also be required to reach the larger proportion of people living in the community who have more common mental disorders such as anxiety or depression, or who have a history of homelessness or contact with the justice system but aren’t currently homeless or in an institution. As these people are less likely to be in touch with services that can deliver programmes directly, strategies with population reach but which are targeted to the concerns and issues faced by people with other disadvantages will also be needed to address the substantially disproportionate burden of smoking that is associated with common mental disorders and socioeconomic disadvantage.

Socioeconomic and psychosocial gradients in smoking are a major contributor to socioeconomic gradients in major health outcomes such as life expectancy and quality of life. The majority of responses to smoking in disadvantaged groups to date have been individually-oriented treatments of tobacco dependence [93]. The factors considered in this paper, common mental disorders and markers of socioeconomic disadvantage, are sufficiently prevalent to suggest policy making and population-based approaches as being a key part of the way forward. This could entail using the tools of population health but adapted to the specific characteristics of these groups, as many of these population groups are too big to reach effectively through individual treatment services.

Monitoring progress in future tobacco control activities, particularly those directed at disadvantaged groups, may require new measurement approaches. For instance, in Australia key indicators are derived from the National Drug Strategy Household Survey [92]. This is a survey conducted by means of a self-complete questionnaire delivered to selected households. The 2010 survey obtained usable responses from around one-third of households selected in the original sample, and under-represented young adults, people who didn’t complete year 12 schooling, single person households, and households from low socioeconomic areas. It is quite likely that differences in methodology, and the low response rate and associated participation biases explain why the estimated proportion of current daily smokers derived from the National Drug Strategy Household Survey (15.1% in the most recent survey in 2010, 16.6% in 2007) is substantially lower than the estimate obtained from face to face surveys with higher response rates [94]. The 2007–08 National Health Survey, conducted by the Australian Bureau of Statistics, estimated 18.9% of Australian adults are current daily smokers [95]. This survey achieved a 91% response rate. Some people with severe forms of disadvantage, such as homeless people, people living in institutions such as correctional facilities or mental health facilities who are known to have very high rates of smoking, are not included in any household surveys.

### Limitations

Not all markers of socioeconomic disadvantage that have been associated with smoking status have been included in this analysis. Because of sample size considerations and in order to preserve the privacy of individual participants in the study, particularly low prevalence demographic indicators were not included on the unit record file released from the NSMHWB. Because of this we were unable to identify in this sample Aboriginal or Torres Strait Islander peoples, pregnant women or people who do not speak English. These groups represent a very small proportion of the NSMHWB sample. Additionally, to preserve the privacy of individual participants in the study, some continuous demographic measures, such as household income and area-level disadvantage, have been categorised on the unit record file released for the survey. Because of this, we were unable to assess whether there could be linear or non-linear associations between these measures of disadvantage and smoking outcomes, or whether the categorical cut-offs provided on the file are optimal for defining disadvantaged groups in respect to smoking behaviours.

As a population-based household interview survey, the NSMHWB was unable to assess low prevalence mental disorders such as schizophrenia or organic psychoses, nor did it include people living in institutional care. Smoking status in Australian adults with psychotic illness has recently been assessed in the second Australian national survey of people living with psychotic illness conducted in 2010 [96]. This survey found that over two-thirds of adults with psychoses were current smokers, unchanged from the first national survey ten years previous [96,97].

Cross-sectional studies, such as the NSMHWB, describe associations but cannot shed light upon causal pathways. The information collected in the survey pertains to current disadvantage or disadvantage in the past 12 months. We don’t have information on the long term accumulation of disadvantage or the intergenerational transfer of disadvantage.

## Conclusions

The association between mental illness and smoking is not explained by the association between mental illness and socioeconomic status. There are strong socioeconomic and psychosocial gradients in both current smoking and smoking cessation. Incorporating knowledge of the other adverse factors in smokers’ lives into tobacco control initiatives may increase the penetration of these interventions in population groups that have historically benefitted less from these activities.

## Competing interests

The authors declare that they have no competing interests.

## Authors’ contributions

DL conceived the original idea for the study. All authors contributed to the development of the study methodology. DL acquired and analysed the data. DL and JH wrote the first draft of the manuscript. All authors edited the paper. All authors read and approved the final manuscript.

## Pre-publication history

The pre-publication history for this paper can be accessed here:

http://www.biomedcentral.com/1471-2458/13/462/prepub

## References

[B1] Australian Bureau of Statistics Health @ a glance, 2011. ABS Cat 4841.0 2011 Canberra: Australian Bureau of Statistics

[B2] Adair T Hoy D Dettrick Z Lopez AD Reconstruction of long-term tobacco consumption trends in Australia and their relationship to lung cancer mortality Cancer Causes Control 2011 22 1047 1053 10.1007/s10552-011-9781-0 21617924

[B3] Burns DM Lee L Shen LZ Gilpin E Tolley HD Vaughn J Shanks TG Burns DM, Garfinkel L, Samet JM Cigarette smoking behavior in the United States Changes in cigarette-related disease risks and their implication for prevention and control 1996 Bethesda: Smoking and Tobacco Control Monograph No. 15, National Cancer Institute 13 112

[B4] Lasser K Boyd JW Woolhandler S Himmelstein DU McCormick D Bor DH Smoking and mental illness. A population-based prevalence study JAMA 2000 284 2606 2610 10.1001/jama.284.20.2606 11086367

[B5] Lawrence D Mitrou F Zubrick SR Smoking and mental illness: results from population surveys in Australia and the United States BMC Publ Health 2009 9 285 10.1186/1471-2458-9-285 PMC2734850 19664203

[B6] Lawrence D Considine J Mitrou F Zubrick SR Anxiety disorders and cigarette smoking: results from the Australian Survey of Mental Health and Wellbeing Aust NZ J Psychiatry 2010 44 520 527 10.3109/00048670903571580 20482412

[B7] Lawrence D Mitrou F Zubrick SR Non-specific psychological distress, smoking status and smoking cessation: United States National Health Interview Survey 2005 BMC Publ Health 2011 11 256 10.1186/1471-2458-11-256 PMC3107796 21513510

[B8] Laaksonen M Rahkonen O Karvonen S Lahelma E Socioeconomic status and smoking: analysing inequalities with multiple indicators Eur J Public Health 2005 15 262 269 10.1093/eurpub/cki115 15755781

[B9] Jarvis M Wardle J Marmot M, Wilkinson R Social patterning of individual health behaviours: the case of cigarette smoking Social determinants of health 1999 New York: Oxford University Press 240 255

[B10] Cavelaars AEJM Kunst AE Geurts JJM Crialesi R Grötvedt L Helmert U Lahelma E Lundberg O Matheson J Mielck A Rasmussen NK Regidor E Spuhler T Mackenbach JP do Rosário-Giraldes M Educational differences in smoking: international comparison BMJ 2000 320 1102 1107 10.1136/bmj.320.7242.1102 10775217 PMC27351

[B11] Lynch JW Kaplan GA Salonen JT Why do poor people behave poorly? Variation in adult health behaviours and psychosocial characteristics by stages of the socioeconomic lifecourse Soc Sci Med 1997 44 809 819 10.1016/S0277-9536(96)00191-8 9080564

[B12] Lantz PM House JS Lepkowski JM Williams DR Mero RP Chen J Socioeconomic factors, health behaviours, and mortality. Results from a nationally representative prospective study of US adults JAMA 1998 279 1703 1708 10.1001/jama.279.21.1703 9624022

[B13] Muntaner C Eaton WW Miech R O’Campo P Socioeconomic position and major mental disorders Epidemiol Rev 2004 26 53 62 10.1093/epirev/mxh001 15234947

[B14] Saraceno B Levav I Kohn R The public mental health significance of research on socio-economic factors in schizophrenia and major depression World Psychiat 2005 4 181 185 PMC1414773 16633546

[B15] Kotz D West R Explaining the social gradient in smoking cessation: it’s not in the trying, but in the succeeding Tob Control 2009 18 43 46 10.1136/tc.2008.025981 18936053

[B16] Crane J Blakely T Hill S Time for major roadworks on the tobacco road? N Z Med J 2004 117 U801 15107904

[B17] Warner K Mendez D Tobacco control policy in developed countries: yesterday, today and tomorrow Nicotine Tob Res 2010 12 867 887 10.1093/ntr/ntq125 20702814

[B18] Emery S Gilpin EA Ake C Farkas AJ Pierce JP Characterizing and identifying “hard-core” smokers: implications for further reducing smoking prevalence Am J Public Health 2000 90 387 394 10705856 10.2105/ajph.90.3.387 PMC1446166

[B19] National Preventative Health Taskforce Technical Report No. 2. Tobacco control in Australia: making smoking history 2008 Canberra: Commonwealth of Australia

[B20] Prochaska JJ Smoking and mental illness—breaking the link N Engl J Med 2011 365 196 198 10.1056/NEJMp1105248 21774707 PMC4457781

[B21] Lawrence D Lawn S Kisely S Bates A Mitrou F Zubrick SR The potential impact of smoke-free facilities on smoking cessation in people with mental illness Aust N Z J Psychiatry 2011 45 1053 1060 10.3109/00048674.2011.619961 22017657

[B22] U.S. Department of Health and Human Services Reducing tobacco use: A report of the Surgeon General 2000 Atlanta, Georgia: U.S. Department of Health and Human Services, Centers for Disease Control and Prevention, National Center for Chronic Disease Prevention and Health Promotion, Office on Smoking and Health

[B23] Australian Bureau of Statistics National Survey of Mental Health and Wellbeing: Summary of Results, 2007. ABS Cat 4326.0 2008 Canberra: Australian Bureau of Statistics

[B24] Slade T Johnston A Teesson M Whiteford H Burgess P Pirkis J Saw S The mental health of Australians 2. Report on the 2007 National Survey of Mental Health and Wellbeing 2009 Department of Health and Ageing: Canberra

[B25] Australian Bureau of Statistics National Survey of Mental Health and Wellbeing: Users’ Guide. ABS Cat 4327.0 2009 Canberra: Australian Bureau of Statistics

[B26] Slade T Johnston A Oakley Browne MA Andrews G Whiteford H 2007 National Survey of Mental Health and Wellbeing: methods and key findings Aust N Z J Psychiatry 2009 43 594 605 10.1080/00048670902970882 19530016

[B27] Kessler RC Ustün TB The World Mental Health (WMH) Survey Initiative version of the World Health Organization (WHO) Composite International Diagnostic Interview (CIDI) Int J Methods Psychiatr Res 2004 13 93 121 10.1002/mpr.168 15297906 PMC6878592

[B28] World Health Organization The ICD-10 classification of mental and behavioural disorders: diagnostic criteria for research 1993 Geneva: World Health Organization

[B29] American Psychiatric Association Diagnostic and statistical manual of mental disorders, fourth edition (DSM-IV) 1994 Washington: American Psychiatric Association

[B30] Australian Bureau of Statistics ANZSCO - Australian and New Zealand Standard Classification of Occupations, first edition, revision 1. ABS Cat 1220.0 2009 Canberra: Australian Bureau of Statistics

[B31] Australian Bureau of Statistics Information paper: an introduction to Socio-Economic Indexes for Areas (SEIFA), 2006 2008 ABS Cat 2039.0. Canberra: Australian Bureau of Statistics

[B32] Wolter K Introduction to variance estimation 2006 New York: Springer

[B33] Rao JNK Scott AJ On simple adjustments to Chi-Square tests with survey data Ann Stat 1987 15 385 397 10.1214/aos/1176350273

[B34] SAS Institute Inc SAS 9.2 Help and Documentation 2002–2009 Cary, NC: SAS Institute Inc

[B35] Evandrou M Falkingham J Smoking behaviour and socio-economic status: a cohort analysis, 1974 to 1998 Health Stat Q 2002 14 30 38

[B36] Hymowitz N Cummings KM Hyland A Lynn WR Pechacek TF Hartwell TD Predictors of smoking cessation in a cohort of adult smokers followed for five years Tob Control 1997 6 Suppl. 2 S57 S62 9583654 10.1136/tc.6.suppl_2.s57 PMC1766209

[B37] Hymowitz N Sexton M Ockene J Grandits G Baseline factors associated with smoking cessation and relapse. MRFIT Research Group Prev Med 1991 20 590 601 10.1016/0091-7435(91)90057-B 1758840

[B38] Lee CW Kahende J Factors associated with successful smoking cessation in the United States, 2000 Am J Public Health 2007 97 1503 1509 10.2105/AJPH.2005.083527 17600268 PMC1931453

[B39] Ziedonis D Hitsman B Beckham JC Zvolensky M Adler LE Audrain-McGovern J Breslau N Brown RA George TP Williams J Calhoun PS Riley WT Tobacco use and cessation in psychiatric disorders: National Institute of Mental Health report Nicotine Tob Res 2008 10 1691 1715 10.1080/14622200802443569 19023823

[B40] Patton GC Hibbert M Rosier MJ Carlin JB Caust J Bowes G Is smoking associated with depression and anxiety in teenagers? Am J Public Health 1996 86 225 230 10.2105/AJPH.86.2.225 8633740 PMC1380332

[B41] Patton GC Carlin JB Coffey C Wolfe R Hibbert M Bowes G Depression, anxiety, and smoking initiation: a prospective study over 3 years Am J Public Health 1998 88 1518 1522 10.2105/AJPH.88.10.1518 9772855 PMC1508459

[B42] Breslau N Peterson E Schultz LR Chilcoat HD Andreski P Major depression and stages of smoking: a longitudinal investigation Arch Gen Psychiatry 1998 55 161 166 10.1001/archpsyc.55.2.161 9477930

[B43] Breslau N Klein DF Smoking and panic attacks: an epidemiologic investigation Arch Gen Psych 1999 56 1141 1147 10.1001/archpsyc.56.12.1141 10591292

[B44] Kendler KS Neale MC Maclean CJ Health AC Eaves LJ Kessler RC Smoking and major depression: a causal analysis Arch Gen Psychiatry 1993 50 36 43 10.1001/archpsyc.1993.01820130038007 8422220

[B45] Brown RA Lewinsohn PM Seeley JR Wagner EF Cigarette smoking, major depression, and other psychiatric disorders among adolescents J Am Acad Child Adolesc Psychiatry 1996 35 1602 1610 10.1097/00004583-199612000-00011 8973066

[B46] Dierker LC Avenevoli S Stolar M Merikangas KR Smoking and depression: an examination of mechanisms of comorbidity Am J Psychiatry 2002 159 947 953 10.1176/appi.ajp.159.6.947 12042182

[B47] Jorm AF Rodgers B Jacomb PA Christensen H Henderson S Korten AI Smoking and mental health: results from a community survey Med J Aust 1999 170 74 77 10026688 10.5694/j.1326-5377.1999.tb126887.x

[B48] Leonard S Adler LE Benhammou K Berger R Breese CR Drebing C Gault J Lee MJ Logel J Olincy A Ross RG Stevens K Sullivan B Vianzon R Virnich DE Waldo M Walton K Freedman R Smoking and mental illness Pharmacol Biochem Behav 2001 70 561 570 10.1016/S0091-3057(01)00677-3 11796154

[B49] Pomerleau OF Pomerleau CS Neuroregulators and the reinforcement of smoking: towards a biobehavioral explanation Neurosci Biobehav Rev 1984 8 503 513 10.1016/0149-7634(84)90007-1 6151160

[B50] Laje RP Berman JA Glassman AH Depression and nicotine: preclinical and clinical evidence for common mechanisms Curr Psychiatry Rep 2001 3 470 474 10.1007/s11920-001-0040-z 11707160

[B51] Glassman AH Cigarette smoking: implications for psychiatric illness Am J Psychiatry 1993 150 546 553 8465868 10.1176/ajp.150.4.546

[B52] Picciotto MR Brunzell DH Caldarone BJ Effect of nicotine and nicotinic receptors on anxiety and depression NeuroReport 2002 13 1097 1106 10.1097/00001756-200207020-00006 12151749

[B53] Pomerleau OF Nicotine as a psychoactive drug: anxiety and pain reduction Psychopharmacol Bull 1986 22 865 869 3797589

[B54] Irvine EE Bagnalasta M Marcon C Motta C Tessari M File SE Chiamulera C Nicotine self-administration and withdrawal: modulation of anxiety in the social interaction test in rats Psychopharmacology 2001 153 315 320 10.1007/s002130000586 11271403

[B55] Hatsukami D Davis GL Wittmers LE al’Absi M Prospective examination of effects of smoking abstinence on cortisol and withdrawal symptoms as predictors of early smoking Drug Alcohol Depend 2004 73 267 278 10.1016/j.drugalcdep.2003.10.014 15036549

[B56] Khantzian EJ The self-medication hypothesis of substance use disorders: a reconsideration and recent applications Harv Rev Psychiatry 1997 4 231 244 10.3109/10673229709030550 9385000

[B57] Williams JM Ziedonis D Addressing tobacco among individuals with a mental illness or an addiction Addict Behav 2004 29 1067 1083 10.1016/j.addbeh.2004.03.009 15236808

[B58] Prochaska JJ Hall SM Tsoh JY Eisendrath S Rossi JS Redding CA Rosen AB Meisner M Humfleet GL Gorecki JA Treating tobacco dependence in clinically depressed smokers: effect of smoking cessation on mental health functioning Am J Public Health 288 98 446 448 17600251 10.2105/AJPH.2006.101147 PMC2253568

[B59] Chapman S Mental health and smoking redux Aust N Z J Psychiatry 2009 42 579 580 19440891 10.1080/00048670902873748

[B60] Chapman S Wakefield M Tobacco control advocacy in Australia: reflections on 30 years progress Health Educ Behav 2001 28 274 289 10.1177/109019810102800303 11380049

[B61] Hill DJ White VM Scollo MM Smoking behaviours of Australian adults in 1995: trends and concerns Med J Aust 1998 168 209 213 9539898 10.5694/j.1326-5377.1998.tb140132.x

[B62] White VM Hayman J Hill DJ Can population-based tobacco-control policies change smoking behaviors of adolescents from all socio-economic groups? Findings from Australia: 1987–2005 Cancer Causes Control 2008 19 631 640 10.1007/s10552-008-9127-8 18264783

[B63] Bank W Curbing the epidemic: Governments and the economics of tobacco control 1999 World Bank: Washington D.C 10.1136/tc.8.2.196 PMC1759728 10478406

[B64] Edwards R ABC of smoking cessation. The problem of tobacco smoking BMJ 2004 328 217 219 10.1136/bmj.328.7433.217 14739193 PMC318495

[B65] Lopez AD Collishaw NE Piha T A descriptive model of the cigarette epidemic in developed countries Tob Control 1994 3 242 247 10.1136/tc.3.3.242

[B66] Ashton M Miller CL Bowden JA Bertossa S People with mental illness can tackle tobacco Aust N Z J Psychiatry 2010 11 1021 1028 21034185 10.3109/00048674.2010.497753

[B67] Williams JM Zimmermann MH Steinberg ML Gandhi KK Delnevo C Steinberg MB Foulds J A comprehensive model for mental health tobacco recovery in New Jersey Adm Policy Ment Health 2011 38 368 383 10.1007/s10488-010-0324-x 21076862 PMC3638154

[B68] Williams JM Dwyer M Verna M Zimmermann MH Gandhi KK Galazyn M Szkodny N Molnar M Kley R Steinberg ML Evaluation of the CHOICES program of peer-to-peer tobacco education and advocacy Community Ment Health J 2011 47 243 251 10.1007/s10597-010-9310-8 20419349

[B69] Ziedonis DM Williams JM Management of smoking in people with psychiatric disorders Curr Opin Psychiatry 2003 16 305 315

[B70] O’Brien J Salmon AM Penman A What has fairness got to do with it? Tackling tobacco among Australia’s disadvantaged Drug Alcohol Rev 2012 31 723 726 10.1111/j.1465-3362.2012.00460.x 22524309

[B71] O’Brien J Bonevski B Salmon A Oakes W Goodger B Seowido D An evaluation of a pilot capacity building initiative for smoking cessation in social and community services: the Smoking Care project Drug Alcohol Rev 2012 31 685 692 10.1111/j.1465-3362.2012.00464.x 22571760

[B72] Bryant J Bonevski B Paul C O’Brien J Oakes W Delivering smoking cessation support to disadvantaged groups: a qualitative study of the potential of community welfare organizations Health Educ Res 2010 25 979 990 10.1093/her/cyq051 20884732

[B73] Bonevski B Paul C D’Este C Sanson-Fisher R West R Girgis A Siahpush M Carter R RCT of a client-centred, caseworker-delivered smoking cessation intervention for a socially disadvantaged population BMC Publ Health 2011 11 70 10.1186/1471-2458-11-70 PMC3038158 21281519

[B74] Bryant J Bonevski B Paul C A survey of smoking prevalence and interest in quitting among social and community service organisation clients in Australia: a unique opportunity for reaching the disadvantaged BMC Publ Health 2011 11 827 10.1186/1471-2458-11-827 PMC3210182 22026718

[B75] Grier S Bryant CA Social marketing in public health Annu Rev Public Health 2005 26 3 19 10.1146/annurev.publhealth.26.021304.144610 15760292

[B76] Le Cook B Wayne GF Keithly L Connolly G One size does not fit all: how the tobacco industry has altered cigarette design to target consumer groups with specific psychological and psychosocial needs Addiction 2003 98 1547 1561 10.1046/j.1360-0443.2003.00563.x 14616181

[B77] Apollonio DE Malone RE Marketing to the marginalised: tobacco industry targeting of the homeless and mentally ill Tob Control 2005 14 409 415 10.1136/tc.2005.011890 16319365 PMC1748120

[B78] Anderson SJ Glantz SA Ling PM Emotions for sale: cigarette advertising and women’s psychosocial needs Tob Control 2005 14 127 135 10.1136/tc.2004.009076 15791023 PMC1748016

[B79] Bloom PN Novelli WD Problems and challenges in social marketing J Marketing 1981 45 79 88 10.2307/1251667 12280283

[B80] Chapman S Falling prevalence of smoking: how low can we go? Tob Control 2007 16 145 10.1136/tc.2007.021220 17565122 PMC2598496

[B81] Durkin S Brennan E Wakefield M Mass media campaigns to promote smoking cessation among adults: an integrative review Tob Control 2012 21 127 138 10.1136/tobaccocontrol-2011-050345 22345235

[B82] Thomas S Fayter D Misso K Ogilvie D Petticrew M Sowden A Whitehead M Worthy G Population tobacco control interventions and their effects on social inequalities in smoking: systematic review Tob Control 2008 17 230 237 10.1136/tc.2007.023911 18426867 PMC2565568

[B83] Farrelly M Bray JW Pechacek T Woollery T Response by adults to increases in cigarette prices by sociodemographic characteristics South Econ J 2001 68 156 165 10.2307/1061518

[B84] Colman G Remler DK Vertical equity consequences of very high cigarette tax increases: if the poor are the ones smoking, how could cigarette tax increases be progressive? NBER working paper series 10906 2004 Cambridge MA: National Bureau of Economic Research

[B85] Warner K The economics of tobacco: myths and realities Tob Control 2000 9 78 89 10.1136/tc.9.1.78 10691761 PMC1748316

[B86] Steptoe A Wardle J Pollard TM Canaan L Davies GJ Stress, social support and health-related behavior: a study of smoking, alcohol consumption and physical exercise J Psychosomatic Res 1996 41 171 180 10.1016/0022-3999(96)00095-5 8887830

[B87] Becker MH Haefner DP Kasl SV Kirscht JP Maiman LA Rosenstock IM Selected psychosocial models and correlates of individual health-related behaviors Med Care 1977 15 27 46 10.1097/00005650-197705001-00005 853780

[B88] Bayer R Stuber J Tobacco control, stigma, and public health: Rethinking the relations Am J Public Health 2006 96 47 50 10.2105/AJPH.2005.071886 16317199 PMC1470446

[B89] Ceci SJ Papierno PB The rhetoric and reality of gap closing. When the “have-nots” gain but the “haves” gain even more Am Psychol 2005 60 149 160 15740447 10.1037/0003-066X.60.2.149

[B90] Niederdeppe J Farrelly M Nonnemaker J Davis KC Wagner L Socioeconomic variation in recall and perceived effectiveness of campaign advertisements to promote smoking cessation Soc Sci Med 2011 72 773 780 10.1016/j.socscimed.2010.12.025 21316830

[B91] Niederdeppe J Fiore M Baker TB Smith SS Smoking-cessation media campaigns and their effectiveness among socioeconomically advantaged and disadvantaged populations Am J Public Health 2008 98 916 924 10.2105/AJPH.2007.117499 18381998 PMC2374829

[B92] Marsh A McKay S Poor smokers 1994 London: Policy Studies Institute

[B93] Warner KE Disparities in smoking are complicated and consequential. What to do about them? Am J Health Promotion 2011 25 S5 S7 10.4278/ajhp.25.5.c3 21510786

[B94] Australian Institute of Health and Welfare 2010 National Drug Strategy Household Survey report 2011 Drug statistics series no. 25. Cat. PHE 145. Canberra: Australian Institute of Health and Welfare

[B95] Australian Bureau of Statistics National Health Survey: Summary of Results 2007–2008 2009 Cat. 4364.0. Canberra: Australian Bureau of Statistics

[B96] Morgan VA Waterreus A Jablensky A Mackinnon A McGrath JJ Carr V Bush R Castle D Cohen M Harvey C Galletly C Stain HJ Neil A McGorry P Hocking B Shah S Saw S People living with psychotic illness 2010 2011 Australian Government Department of Health and Ageing: Canberra 10.1177/0004867412449877 22696547

[B97] Cooper J Mancuso SG Borland R Slade T Galletly C Castle D Tobacco smoking among people living with a psychotic illness: the second Australian survey of psychosis Aust N Z J Psychiatry 2012 46 851 863 10.1177/0004867412449876 22645396

